# Supplementary material for: Multi‐dataset identification of innovative feature genes and molecular mechanisms in keratoconus
Source: J Cell Mol Med. 2024 Sep 19;28(18):e70079. doi: 10.1111/jcmm.70079 (PMC11412914; doi:10.1111/jcmm.70079)
Supplement: Supplementary file 2 — Material S2. [file JCMM-28-e70079-s002.zip › 7.GSEA analysis/4.MYRF/KEGG_MISMATCH_REPAIR.html]

Details for gene set KEGG\_MISMATCH\_REPAIR[GSEA]

|  || Dataset | MYRF.group.cls#h\_versus\_l |
| Phenotype | group.cls#h\_versus\_l |
| Upregulated in class | h |
| GeneSet | KEGG\_MISMATCH\_REPAIR |
| Enrichment Score (ES) | 0.24297099 |
| Normalized Enrichment Score (NES) | 0.83801275 |
| Nominal p-value | 0.74385244 |
| FDR q-value | 1.0 |
| FWER p-Value | 1.0 |
Table: GSEA Results Summary

  

Fig 1: Enrichment plot: KEGG\_MISMATCH\_REPAIR      
 Profile of the Running ES Score & Positions of GeneSet Members on the Rank Ordered List

  

| SYMBOL | TITLE | RANK IN GENE LIST | RANK METRIC SCORE | RUNNING ES | CORE ENRICHMENT || 1 | RPA4 | na | 616 | 0.541 | 0.2430 | Yes |
| 2 | PMS2 | na | 10036 | 0.095 | 0.0175 | No |
| 3 | MSH3 | na | 11107 | 0.076 | 0.0236 | No |
| 4 | SSBP1 | na | 13497 | 0.044 | -0.0241 | No |
| 5 | POLD1 | na | 14812 | 0.030 | -0.0478 | No |
| 6 | MLH3 | na | 15678 | 0.021 | -0.0625 | No |
| 7 | RPA1 | na | 16107 | 0.017 | -0.0668 | No |
| 8 | POLD4 | na | 17303 | 0.006 | -0.0983 | No |
| 9 | MSH2 | na | 18082 | -0.001 | -0.1202 | No |
| 10 | PCNA | na | 18478 | -0.005 | -0.1292 | No |
| 11 | POLD2 | na | 20484 | -0.027 | -0.1737 | No |
| 12 | RPA2 | na | 20868 | -0.032 | -0.1694 | No |
| 13 | MSH6 | na | 21088 | -0.035 | -0.1590 | No |
| 14 | POLD3 | na | 22606 | -0.054 | -0.1769 | No |
| 15 | RPA3 | na | 23148 | -0.062 | -0.1626 | No |
| 16 | MLH1 | na | 23522 | -0.068 | -0.1406 | No |
| 17 | LIG1 | na | 23679 | -0.070 | -0.1111 | No |
| 18 | RFC5 | na | 23917 | -0.074 | -0.0822 | No |
| 19 | RFC1 | na | 25754 | -0.108 | -0.0832 | No |
| 20 | RFC2 | na | 26486 | -0.123 | -0.0450 | No |
| 21 | RFC4 | na | 26832 | -0.131 | 0.0082 | No |
| 22 | RFC3 | na | 26843 | -0.131 | 0.0711 | No |
| 23 | EXO1 | na | 32108 | -0.324 | 0.0755 | No |
Table: GSEA details [plain text format]

  

Fig 2: KEGG\_MISMATCH\_REPAIR      
 Blue-Pink O' Gram in the Space of the Analyzed GeneSet

  

Fig 3: KEGG\_MISMATCH\_REPAIR: Random ES distribution      
 Gene set null distribution of ES for **KEGG\_MISMATCH\_REPAIR**
